# SAPHO syndrome: the supposed trigger by isotretinoin, the efficacy of adalimumab and the specter of depressive disorder: a case report

**DOI:** 10.1186/s13052-020-00933-1

**Published:** 2020-11-13

**Authors:** Michele Luzzati, Gabriele Simonini, Cesare Filippeschi, Teresa Giani, Sandra Trapani

**Affiliations:** 1 Post-Graduate School of Pediatrics, University of Florence, Florence, Italy; 2 Department of NEUROFARBA, University of Florence, Florence, Italy; 3 Rheumatology Department, Meyer Children’s University Hospital, Florence, Italy; 4 Dermatology Department, Meyer Children’s University Hospital, Florence, Italy; 5 Department of Health Science, University of Florence, Florence, Italy

**Keywords:** Isotretinoin, Depression, Adalimumab, Osteitis, Hidradenitis suppurativa, Acne fulminans

## Abstract

**Background:**

SAPHO (synovitis, acne, pustolosis, hyperostosis and osteitis) syndrome is a rare autoinflammatory chronic disorder, presenting with non-infectious osteitis, sterile joint inflammation and skin manifestations including palmoplantar pustolosis and severe acne.

It could be often misdiagnosed for its heterogeneous clinical presentation. Treatment is challenging and, due to the rarity of this syndrome, no randomized controlled clinical trials have been conducted. Empirical treatments, including non-steroidal anti-inflammatory drugs (NSAIDs), corticosteroids, antibiotics and bisphosphonates and disease-modifying anti-rheumatic drugs (DMARDs) could be quite effective. Anti-tumor necrosis factor-alpha (anti-TNF-α) agents and interleukin-1 (IL-1) antagonists have shown promising results in refractory patients. Isotretinoin, commonly used for severe acne, has been rarely described as possible trigger of osteo-articular manifestations, in particular sacroiliitis.

**Case presentation:**

The case of a boy, affected by acne fulminans and depression, who presented with sacroiliitis after a 10-week treatment with isotretinoin is presented. After SAPHO diagnosis, NSAIDs therapy was started but the onset of bilateral gluteal hidradenitis suppurativa required the switch to a TNF-α antagonist (Adalimumab) with the achievement of a good control of the disease. Despite specific therapy with sertraline, the patient continued to complains severe depression.

**Conclusions:**

Our case reports a temporal association between the onset of osteo-articular symptoms and the introduction of isotretinoin, as previously described. However, this timeline is not sufficient to establish a causal role of this drug into the pathogenesis of sacroiliitis. At this regard, further studies are required. The occurrence of hidradenitis suppurativa during SAPHO course supported the introduction of TNF-α blockers with a favourable result, as reported in a few cases in literature. The association between SAPHO syndrome and depressive mood disorders is already reported. Our patient experienced severe depression whose trend seems to be independent from the course of the main disease. Currently, it is not clarified if depression could be considered reactive to the underling disease or if it forms an integral part of the autoinflammatory disorder.

## Background

Synovitis Acne Pustulosis Hyperostosis Osteitis (SAPHO) syndrome is an uncommon disease, characterized by chronic inflammatory osteoarticular and dermatological lesions. Its prevalence (< 1 in 10.000) is recognised as being underestimated [1]. The SAPHO syndrome is often misdiagnosed for its heterogeneous clinical presentation. Anterior chest wall and axial skeleton joints are the most commonly involved osteoarticular sites. Cutaneous manifestations include various acneiform and neutrophilic dermatoses: palmoplantar pustolosis is the most commonly reported lesion; moderate to severe acne, including acne conglobate and acne fulminans, and hidradenitis suppurativa (HS) occur approximately in 25% of patients [2]. Finally, patients may have the potential to develop depressive symptoms [3].

The pathogenesis of SAPHO syndrome is not defined, but likely includes a combination of genetic, infectious and immunological components leading to the activation of innate and cell-mediate immune system [4]. Therefore, it is classified as an autoinflammatory disorder [5].

Isotretinoin, representing the first-choice treatment for severe acne in adolescents, has been reported as potential trigger of osteoarticular symptoms, although its pathogenesis remains unclear. Conversely, osteoarticular involvement triggered by isotretinoin has been very rarely described in SAPHO [6]. Since SAPHO syndrome is rare and no therapeutic trials are available, the therapy is aimed to modify the inflammatory process and control symptoms. Conventional first-line treatments include various drugs such as NSAIDs, corticosteroids, DMARDs and bisphosphonates. Moreover, the use of anti-TNF agents has been proved to be a valid therapeutic regimen for unresponsive cases [7]. The use of adalimumab (ADA), a tumour necrosis factor (TNF)-α antagonist, has been proved to be a valid therapeutic regimen for cases of refractory SAPHO or complicated with HS [8]. Recently, non-anti-TNF- α agents have revealed as effective alternative treatments. Tocilizumab has been proposed for its known activity on osteoclasts and Ustekinumab for its efficacy against palmoplantar psoriasis [9].

We report a case of severe SAPHO syndrome complicated by HS, successfully treated with ADA. Despite the good control of disease, a depressive mood disorder persisted.

## Case presentation

A Caucasian 15-year old male complained of severe acne and low back pain with inability to walk. His previous medical history was unremarkable except for the onset of acne vulgaris during his puberty. Three months before admission, he had showed a dramatic worsening of acne characterized by several cystic skin lesions with extensive ulcerating and inflammatory components, assuming a form of acne fulminans. Therefore, a course of systemic isotretinoin therapy was administered at the dose of 0.5 mg/kg/daily without any benefit. In addition, the boy developed depressive symptoms associated with insomnia and irritability. Thus, sertraline therapy was started.

**Fig. 1:**
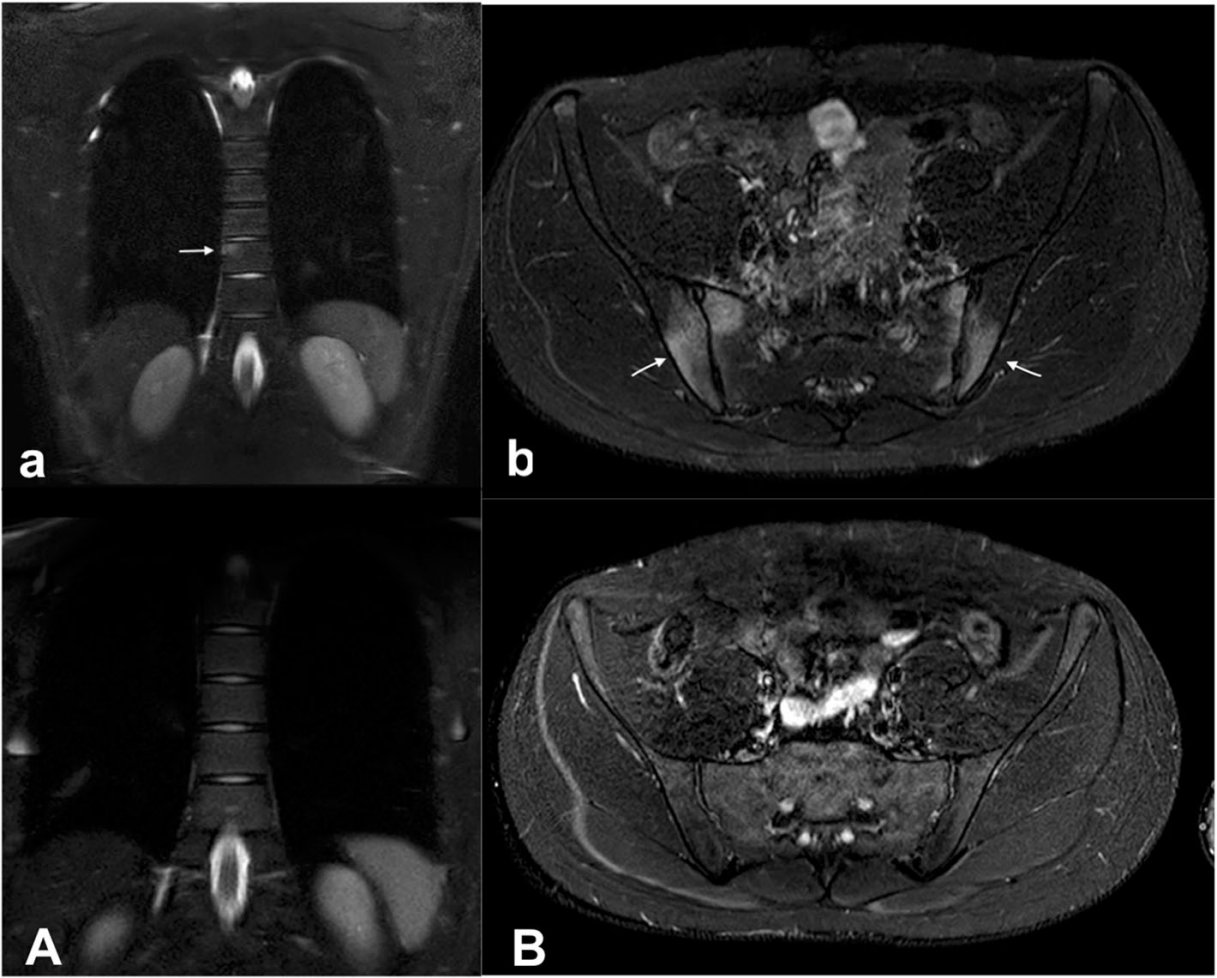
MRI STIR images of lumbar osteitis and sacroiliac involvement before (upside) and after ADA treatment (downside)

**Fig. 2:**
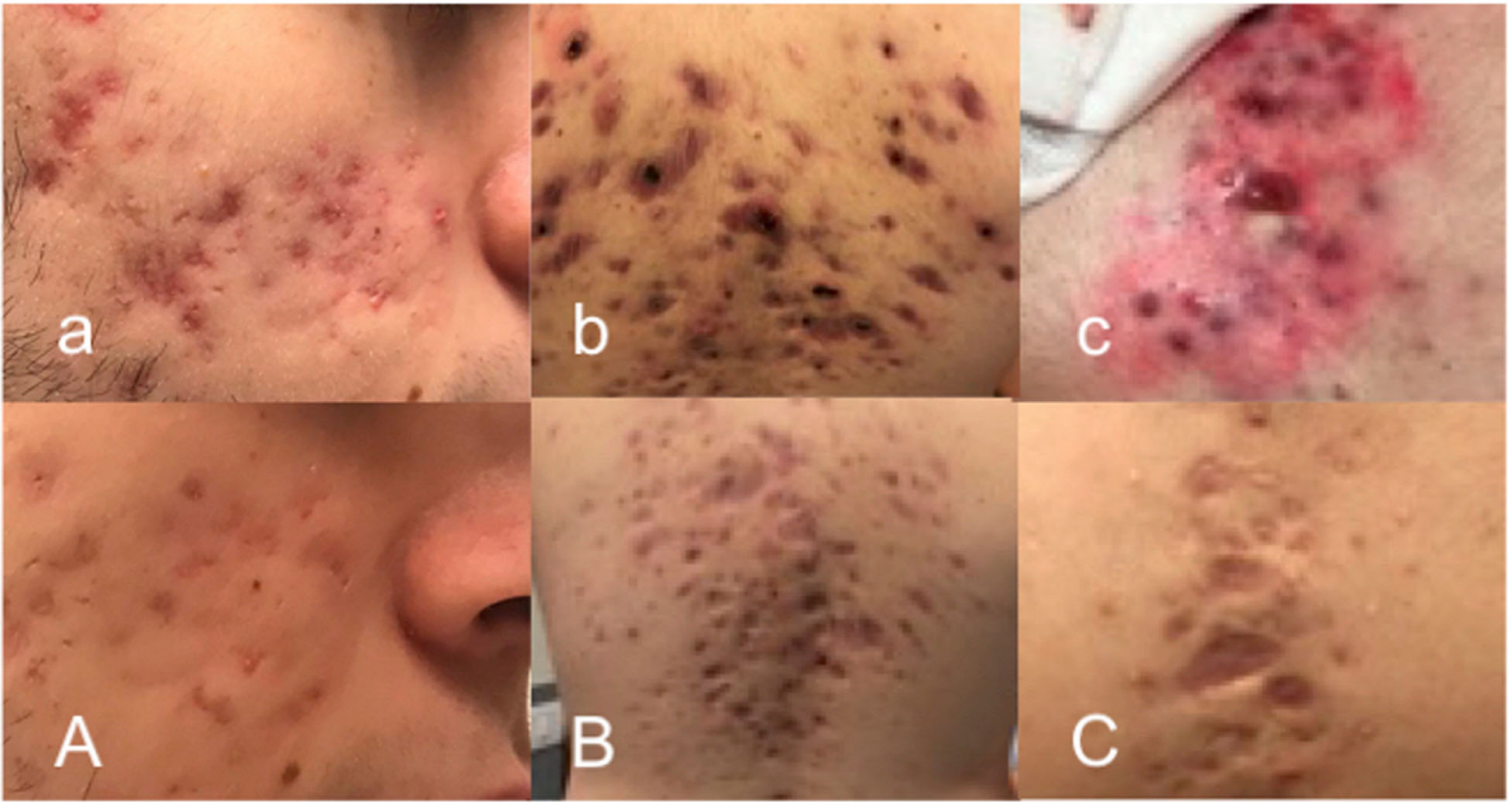
Cutaneous lesions on the face, back and sternal region before (upside) and after (downside) ADA treatment

Ten weeks after starting isotretinoin, the patient experienced increasing low-back pain with progressive inability to walk and restriction of daily activities. At admission to the emergency department, he was suffering and unable to walk. Physical examination revealed a decreased axial range of movement, sacroiliac pain and severe nodulocystic acne with abscesses on face, neck and thorax. Laboratory investigations revealed systemic inflammation (CPR 7.15 mg/dl [normal range < 0.5 mg/dl], ESR 84 mm/h [normal range < 30 mm/h], WBC 14,950/mmc, 70% neutrophils). All rheumatologic parameters including complement and autoantibodies were within normal range. The patient tested negative for HLA-B27 typing. Culture of the pustular lesions was positive for *Staphylococcus aureus*. Antibiotic therapy with clindamycin was introduced and isotretinoin was interrupted. Pelvis and hip X-ray was unremarkable. Magnetic resonance imaging (MRI) showed moderate bone marrow oedema and osteitis of transverse process of fifth lumbar vertebra (Fig. 1a) and symmetrical sacroiliitis (Fig. 1b). The association of acne *fulminans* and osteitis suggested the diagnosis of SAPHO syndrome. Whole body MRI confirmed this hypothesis revealing anterior chest wall, in particular coronal views showed sternoclavicular and costoclavicular osteitis, and axial involvement, including sacroiliac joint and spine. Consequently, intravenous antibiotics were interrupted and, NSAIDs and oral rifampicin were started, in order to control the inflammatory status and the cutaneous lesions, respectively. Despite the specific treatment, the depressive disorder was persisting along with the other complaints. Nevertheless, during the following weeks osteoarticular involvement and cutaneous manifestations worsened and gluteal bilateral HS appeared. Therefore, ADA was subcutaneously administered at the dosage of 40 mg/dose every 2 weeks. After 4 weeks of treatment a progressive improvement both of cutaneous lesions and osteoarticular symptoms was reported and ADA administration interval (at the same dose of 40 mg) was extended to 4 weeks. After six months of favourable ADA treatment, the boy experimented a relapse of osteoarticular symptoms. Therefore, ADA was administered again every 2 weeks obtaining a long-lasting remission. An MRI performed 12 months later, has shown no evidence of abnormal vertebral bone marrow signal (Fig. 1a), sterno-clavear or sacroiliac effusions (Fig. 1b). After 24-months treatment with ADA, the disease maintains complete remission. In Fig. 2 we show cutaneous lesions on the face, on the back and on the sternal region before and after ADA treatment. On the contrary, the depressive mood disorder persists and negatively affects the quality of patient’s life.

## Discussion and conclusions

SAPHO syndrome is an uncommon auto inflammatory disease. Its pathogenesis could be the consequence of the complex interactions between polygenic and exogenous factors triggering the disease burden [2].

In our case acne fulminans had preceded the onset of sacroiliitis. As previously reported skin manifestations may precede the onset of osteoarticular symptoms in 30 to 50% of patients [5, 10].

However, a few cases documented that isotretinoin was as a possible causing factor for articular symptoms. Two different case reports described the development of symmetrical sacroiliitis in two boys one and three months after the introduction of isotretinoin. Their symptoms improved with the concurrent isotretinoin interrumption and NSAIDs starting [11, 12]. Furthermore, *Coskun *et al. in a review of 21 cases concluded that sacriliitis could be a rare complication of isotretinoin treatment [13].

Previous studies supported the hypothesis that isotretinoin has detergent-like properties and could alter cell membrane structures, promoting the degenerative process of joints [14]. Moreover, retinol and acid retinoic has been shown to induce metalloproteinases activity, which increase degradation of membranes [6, 14, 15].

However, isotretinoin remains a valuable and effective treatment of severe acne, its use is supported in combination with corticosteroids also to treat acne fulminans [16].

Our patient experienced osteoarticular symptoms 10 weeks after being on isotretinoin. Indeed, only a temporal association between the onset of osteo-articular symptoms and the introduction of this drug has been demonstrated. Moreover, SAPHO syndrome may present a remitting and relapsing course, and skin manifestations could precede osteoarticular symptoms in almost an half of cases. Hence, these timeline is not sufficient to establish a causal role of this drug into the pathogenesis of sacroiliitis.

Considering that the available data about the role of isotretinoin regard a small sample of patients, further studies are required. Awaiting for new evidences, we suggest a wise approach in case of adolescents suffering from acne and osteoarticular symptoms, particularly with axial skeleton involvement, in order to examine a trigger role of previous isotretinoin administration.

In our case due to the onset of HS we decide to introduce ADA treatment. HS is a chronic, suppurative skin manifestantion associated to SAPHO syndrome. It is characterized by the involvement of the apocrine glands, in particular of the axillae and groin.

To our knowledge there are only few cases reporting a favourable effect of ADA in the treatment of HS associated to SAPHO syndrome [17–19]. ADA is considered the first-choice biologic agent in moderate/severe HS after failure of conventional treatments and in refractory SAPHO syndrome [8, 17]. Our patient experimented a rapid clinical and radiological improvement with ADA administration every 2 weeks at 12 and 24 month follow-up period.

Unfortunately, the boy who had complained depression since the worsening of acne, still maintained a severe psychiatric disorder in the following years, despite the sertraline therapy and the dermatological and rheumatological stable remission.

The association between many immuno-mediated inflammatory diseases and depressive mood disorders is already reported [20]. There is no related literature concerning psychiatric symptoms in SAPHO patients, out of a recent study by Lu et al., revealing a high prevalence (46%) of depression in SAPHO patients. Twenty-eight SAPHO patients underwent to psychiatric evaluation and MRI scans. The study confirmed the great prevalence of depressive mood disorders and revealed an abnormal brain activity at functional MRI sequences suggesting that SAPHO patients may potentially develop depression.

Considering the psychological impact that skin manifestations could have especially during adolescence it is not clarified if depression could be considered reactive to the underling disease or if it forms an integral part of the autoinflammatory disorder.

In conclusion, the temporal association between isotretinoin and sacroiliitis has been already described but a real pathogenetic mechanism is not proved. In severe cases of SAPHO syndrome Adalimumab therapy demonstrated persistent clinical remission, even in cases complicated by HS. SAPHO patients may develop depressive symptoms, but further studies should investigate its pathogenesis.

## Data Availability

Not applicable.

## Abbreviations

NSAIDs: Nonsteroidal anti-inflammatory drugs
HS: Hidradenitis suppurativa
ADA: Adalimumab
DMARDs: Disease modifying antirheumatic drugs
CRP: C-Reactive protein
ESR: Erythrocyte sedimentation rate
WBC: White blood cells
MRI: Magnetic resonance imaging
TNF: Tumour necrosis factor
IL: Interleukin
